# Neoadjuvant ctDNA-MRD monitoring uncovers occult progression despite radiologic response in locally advanced hypopharyngeal carcinoma: a case report

**DOI:** 10.3389/fonc.2026.1748855

**Published:** 2026-03-27

**Authors:** Zhaoyang Wang, Lin Gui, Ye Zhang, Shengkai Huang, Shun Wang, Xiwei Zhang, Fa Zhang, Xiaotong Yan, Zongmin Zhang, Shaoyan Liu, Changming An

**Affiliations:** 1 Department of Head and Neck Surgery, National Cancer Center/National Clinical Research Center for Cancer/Cancer Hospital, Chinese Academy of Medical Sciences and Peking Union Medical College, Beijing, China; 2 Department of Medical Oncology, National Cancer Center/National Clinical Research Center for Cancer/Cancer Hospital, Chinese Academy of Medical Sciences and Peking Union Medical College, Beijing, China; 3 Department of Radiation Oncology, National Cancer Center/National Clinical Research Center for Cancer/Cancer Hospital, Chinese Academy of Medical Sciences and Peking Union Medical College, Beijing, China; 4 Department of Clinical Laboratory, National Cancer Center/National Clinical Research Center for Cancer/Cancer Hospital, Chinese Academy of Medical Sciences and Peking Union Medical College, Beijing, China; 5 Department of Pathology, National Cancer Center/National Clinical Research Center for Cancer/Cancer Hospital, Chinese Academy of Medical Sciences and Peking Union Medical College, Beijing, China

**Keywords:** CtDNA, hypopharyngeal carcinoma, immunochemotherapy, MRD, neoadjuvant therapy, treatment response

## Abstract

**Background:**

The assessment of treatment response during neoadjuvant therapy for head and neck squamous cell carcinoma (HNSCC) relies largely on radiographic and endoscopic evaluations, which may fail to detect minimal residual disease (MRD). Circulating tumor DNA (ctDNA)-based MRD monitoring has emerged as a sensitive biomarker capable of revealing molecular disease activity that is not captured by imaging. Evidence supporting its use in the neoadjuvant setting of HNSCC remains limited.

**Case presentation:**

We report a 53-year-old man with locally advanced hypopharyngeal squamous cell carcinoma (cT4N2cM0) who received three cycles of neoadjuvant immunochemotherapy (toripalimab, cisplatin, and nab-paclitaxel). Radiologic and endoscopic assessment demonstrated a partial response with a 71.6% reduction in tumor burden and near-complete mucosal normalization. In contrast, ctDNA-MRD analysis showed elevated ctDNA levels compared with baseline, indicating molecular progression. Despite the favorable imaging response, the patient developed new distant metastases three months after definitive chemoradiotherapy and maintenance immunotherapy, confirming systemic disease progression.

**Discussion:**

This case highlights the limitations of imaging in differentiating true tumor regression from residual disease during neoadjuvant therapy. Persistent ctDNA-MRD despite radiologic remission may signal occult progression and could influence critical treatment decisions, including the choice between surgery, chemoradiation, or systemic intensification. Accumulating evidence from other tumor types suggests that MRD clearance may predict pathological complete response (pCR), raising the possibility that accurate molecular assessment could help identify patients who may safely avoid surgery. However, MRD applications in neoadjuvant HNSCC—particularly hypopharyngeal carcinoma—remain sparsely studied.

**Conclusion:**

ctDNA-MRD may provide complementary and potentially earlier insights into treatment response compared with imaging alone. Its incorporation into neoadjuvant strategies for HNSCC warrants further investigation and may ultimately support more personalized and accurate therapeutic decision-making.

## Introduction

Head and neck squamous cell carcinoma (HNSCC) remains a biologically heterogeneous disease with highly variable responses to neoadjuvant therapy. Although radiographic and endoscopic assessments are routinely used to evaluate treatment efficacy, these methods may miss minimal residual disease (MRD) and fail to capture early molecular signs of progression. Circulating tumor DNA (ctDNA) has emerged as a sensitive biomarker capable of reflecting real-time changes in tumor burden, detecting occult disease, and predicting relapse earlier than conventional imaging (1).

While the analytical and clinical validity of ctDNA-based MRD assessment is supported by a growing body of evidence across solid tumors, its clinical utility—particularly in guiding treatment decisions in the neoadjuvant setting—remains to be fully established (2). In head and neck cancer, where response evaluation directly influences the choice between surgery, definitive chemoradiotherapy, or systemic treatment escalation, more precise tools beyond conventional imaging are needed.

This case highlights a marked discordance between radiologic remission and ctDNA-MRD dynamics during neoadjuvant treatment, underscoring the ability of molecular monitoring to uncover early disease persistence that may not be apparent on imaging.

## Case description

A 53-year-old male presented in November 2023 with a 6-month history of pharyngeal discomfort and progressive hoarseness and dyspnea for 2 months. Due to worsening airway obstruction, an emergency tracheostomy was performed at an outside hospital. On presentation to our center, physical examination revealed hoarseness and bilateral fixed, hard cervical lymph nodes (right: ~3 cm, left: ~2 cm).

**Figure 1:**
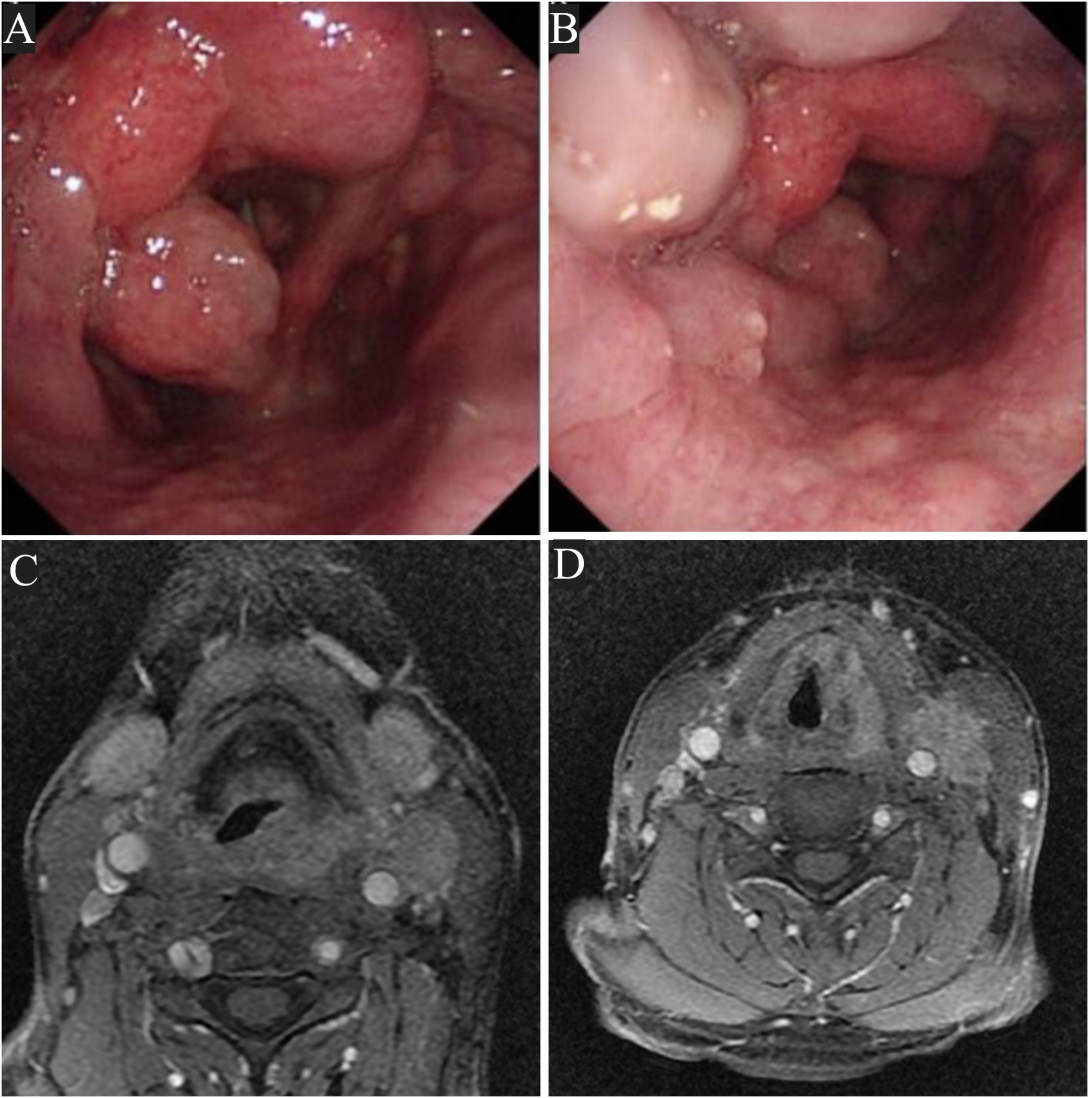
Pre-treatment laryngoscopy **(A, B)** and contrast-enhanced MRI **(C, D)**. This demonstrated a hypopharyngeal carcinoma with extensive involvement of the left pharyngolaryngeal region, accompanied by bilateral cervical lymph node metastases.

Laryngoscopy revealed a cauliflower-like mass occupying the left pyriform sinus, invading the left aryepiglottic fold, pharyngoepiglottic fold, arytenoid, ventricular band, epiglottic lingual surface, and left hemilarynx. The left vocal cord was fixed, while the right vocal cord maintained normal mobility. The epiglottic lingual surface and hypopharyngeal posterior wall could not be adequately visualized due to tumor obstruction. The right pyriform sinus appeared normal, and no abnormalities were noted in the nasopharynx (**Figure 1**).

Neck ultrasonography (B-US) indicated suspected metastases in bilateral level II, III, and IV lymph nodes.

Contrast-enhanced CT and MRI both demonstrated a malignant-appearing mass involving the pharyngolaryngeal region, with extension into the left paraglottic space, anterior commissure, and thyroid cartilage. The lesion measured approximately 3 cm in maximal dimension, showed heterogeneous enhancement, and exhibited restricted diffusion on MRI. Multiple enlarged left retropharyngeal and bilateral cervical level II–IV lymph nodes were present, with the largest measuring approximately 1.8 cm in short axis (**Figure 1**). No obvious primary lesions were found in the nasopharynx, salivary glands, or thyroid gland. A small solid nodule (0.4 × 0.3 cm) was noted in the right lower lung lobe; multiple subpleural fibrotic strands (predominantly left) were present. The liver contained several low-density foci (largest 0.5 cm), the gallbladder harbored multiple stones, and the left kidney exhibited a 1.6 × 1.4 cm hypodense lesion—all considered benign or indeterminate at this stage.

Histopathologic examination of the biopsy specimen revealed a moderately to poorly differentiated squamous cell carcinoma. Immunohistochemical staining demonstrated p16 (3+), p53 (20%+), PD-L1 combined positive score (CPS) of 10, Ki-67 index of 70%, along with EGFR (2+), VEGF (-), HER2 (1+) and EBER (-). Integrating the clinical presentation, radiologic findings, and pathological results, the case was diagnosed as hypopharyngeal squamous cell carcinoma, classified as cT4N2cM0 according to the AJCC 8th edition.

From December 2023 to February 2024, the patient received three cycles of neoadjuvant immunochemotherapy (toripalimab 240 mg on day 1, cisplatin 140 mg on day 1, and nab-paclitaxel 270 mg on day 1; every 3 weeks). Treatment was administered within the framework of a prospective clinical trial (3). Although two cycles were predefined in the study protocol, a third cycle was administered based on good tolerability and investigator discretion. The regimen was well tolerated, with only grade 1–2 fatigue and nausea, and no immune-related adverse events were observed.

**Figure 2:**
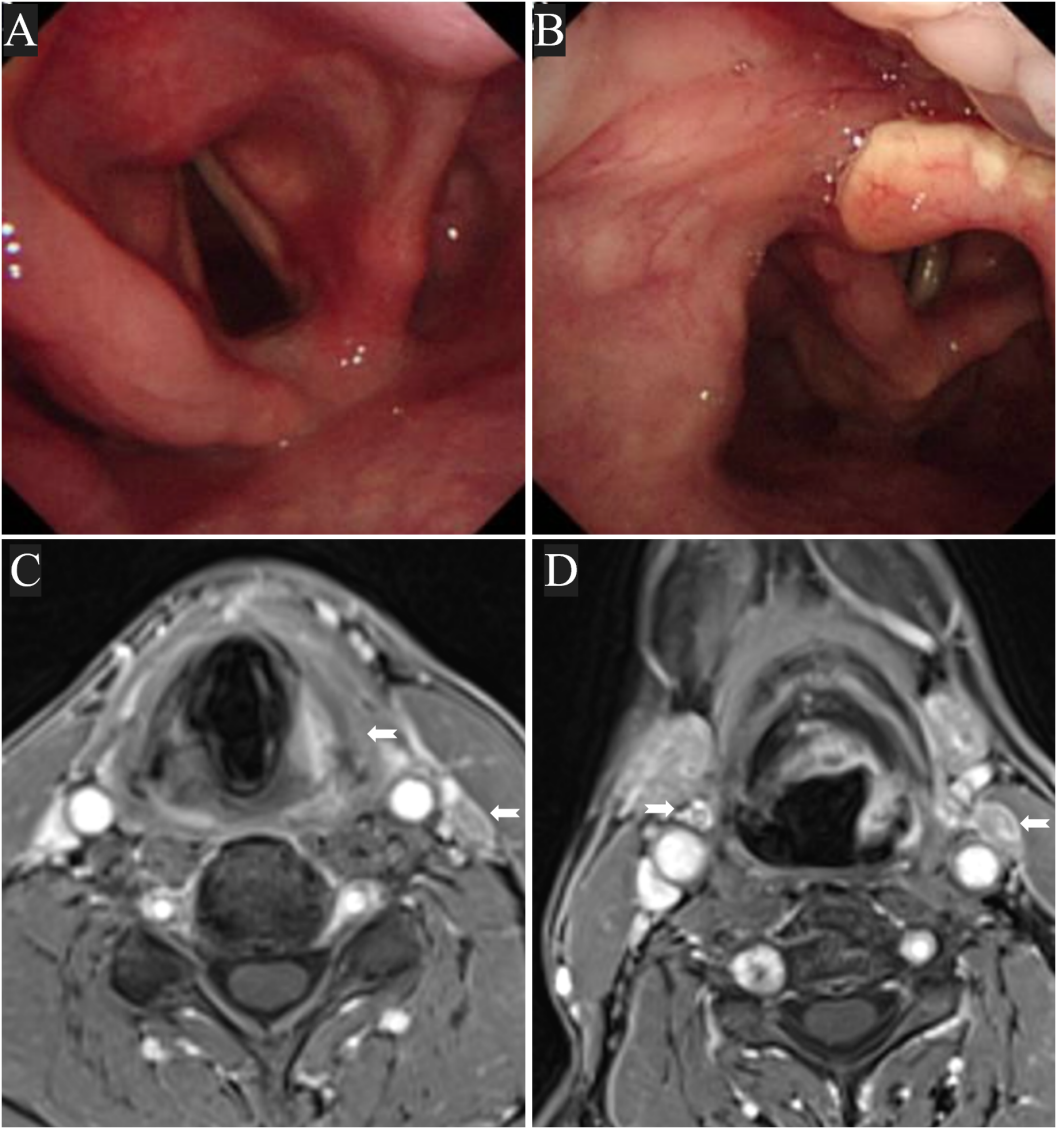
Laryngoscopy **(A, B)** and MRI **(C, D)** after neoadjuvant therapy. There was a marked tumor reduction (71.6% reduction in tumor volume), with no visible residual tumor on endoscopy. White arrows indicate areas of residual signal abnormality on MRI.

Response assessment was performed in February 2024, after completion of three cycles neoadjuvant therapy. CT and MRI showed a partial response (PR) with a 71.6% reduction in measurable tumor volume according to RECIST 1.1 criteria. Endoscopic examination demonstrated a nearly flattened mucosal surface with no visible residual tumor, indicating an excellent clinical response (**Figure 2**).

**Figure 3:**
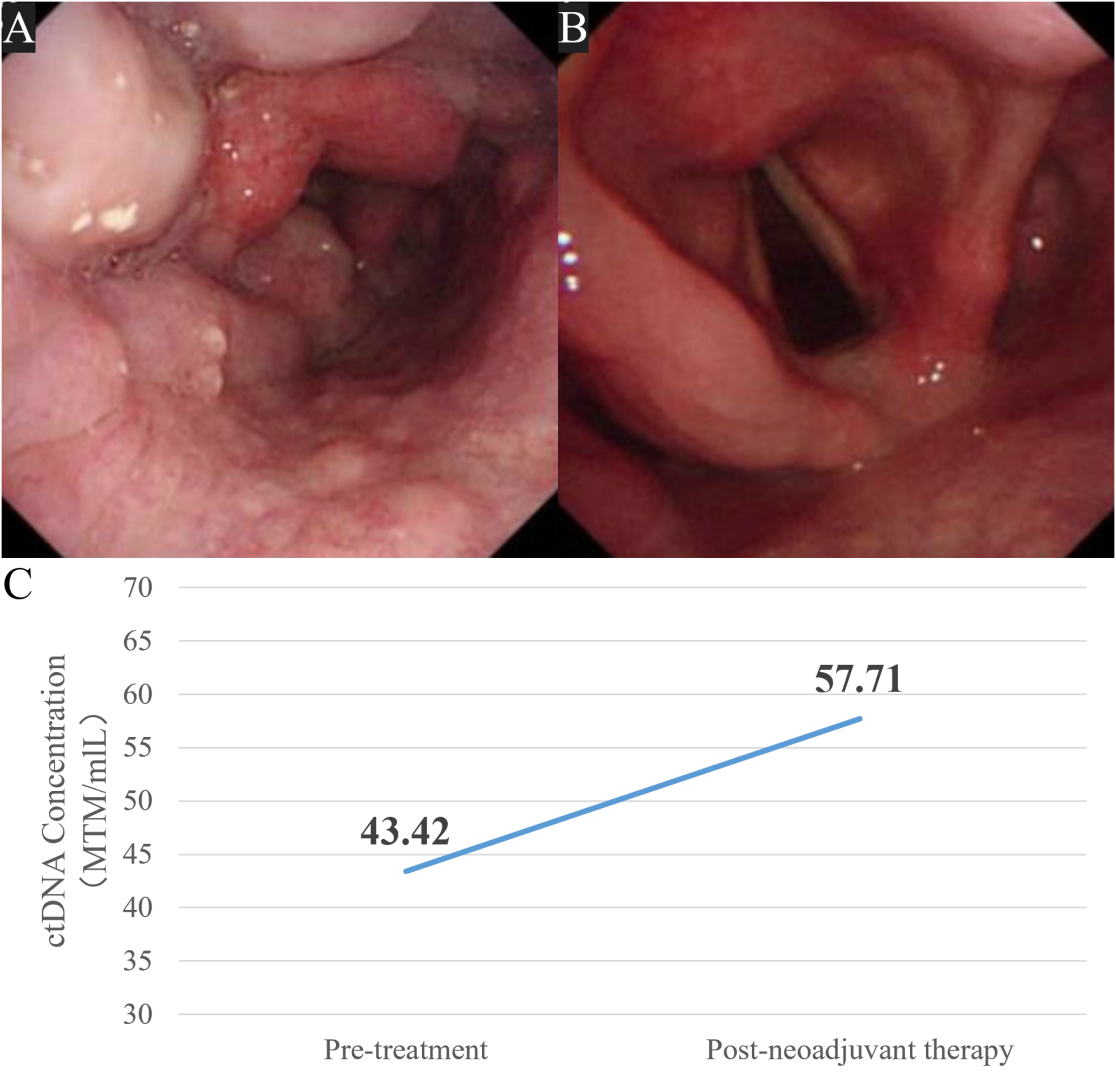
Baseline **(A)** and post-neoadjuvant therapy **(B)** laryngoscopy, with corresponding ctDNA-MRD dynamics **(C)**. This illustrated the discordance between ctDNA-MRD trends and imaging findings, with MRD elevation despite apparent radiologic improvement.

However, ctDNA-MRD analysis performed at the same timepoint showed a notable increase in plasma ctDNA compared with baseline, indicating potential molecular progression despite the radiologic remission. A tumor-naïve assay was used, in which baseline peripheral blood cell-free DNA underwent genomic profiling using the OncoD-P1021B panel (GenePlus^®^, covering 1, 021 cancer-related genes), followed by customized probe–based high-throughput MRD detection (Onco1021-MRD-B, GenePlus^®^). Two mutations were identified in peripheral blood at baseline: EPHA7 NM_004440.3 c.2630G>A (p.R877H) and TBX3 NM_016569.3 c.1132G>A (p.E378K). To exclude variants arising from clonal hematopoiesis of indeterminate potential (CHIP), paired sequencing of peripheral blood mononuclear cells (PBMCs) was performed, and only tumor-associated variants were retained for MRD assay design. The ctDNA concentration increased from 43.42 MTM/ml at baseline to 57.71 MTM/ml after treatment, consistent with molecular disease persistence or progression (**Figure 3**). MTM/ml (mean tumor molecules per milliliter) represents the quantified number of tumor-derived DNA molecules per milliliter of plasma, as defined by the assay platform. ctDNA analyses were performed for research purposes, and results were not disclosed to the treating clinicians during therapeutic decision-making.

**Figure 4:**
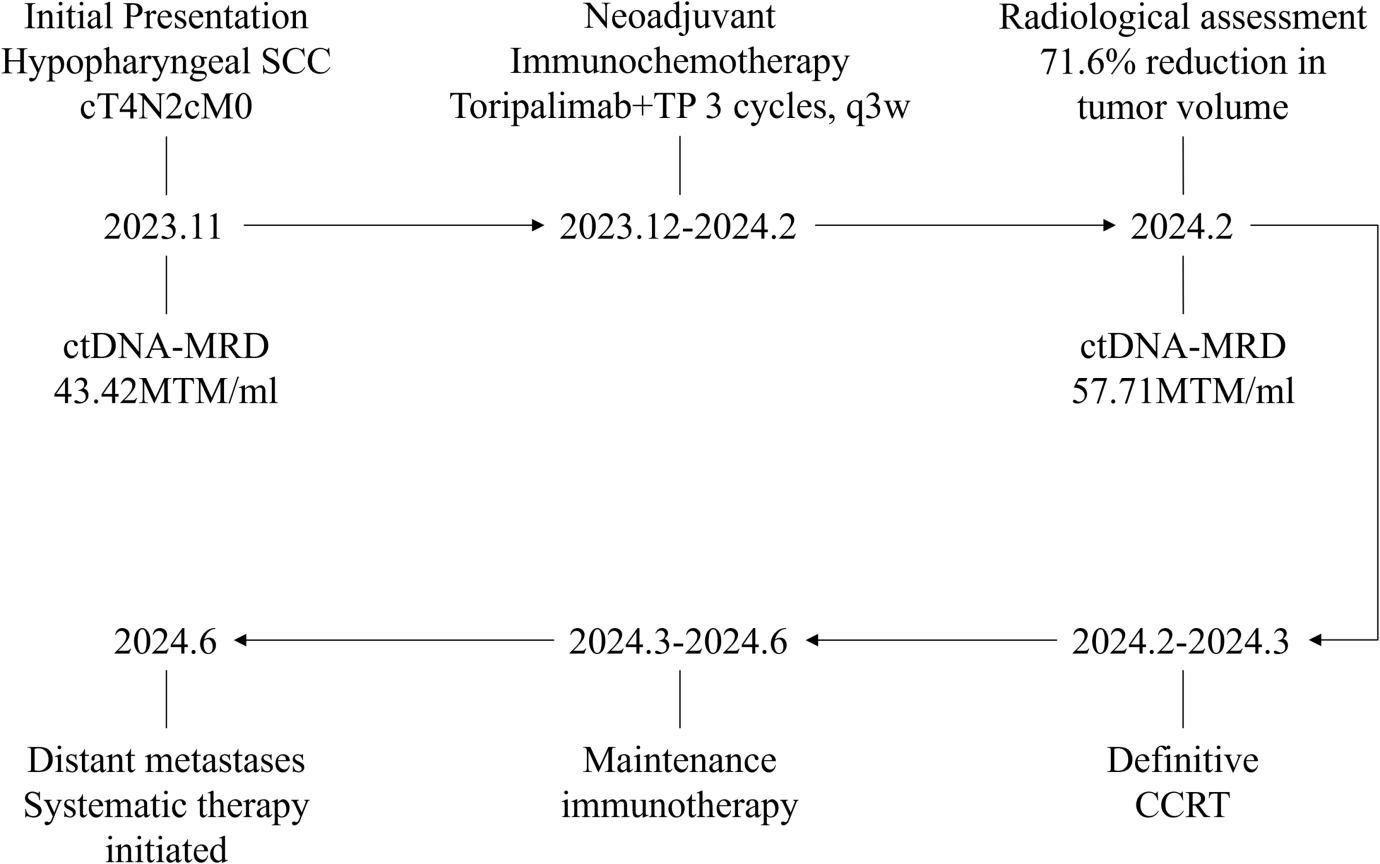
Clinical timeline and dynamic changes in ctDNA-MRD.

Given the apparent clinical response, the patient proceeded to definitive concurrent chemoradiotherapy (CCRT), consisting of a total dose of 70 Gy with concurrent cisplatin 150 mg every 3 weeks. Definitive CCRT was initiated in February 2024 and completed in March 2024. After completing CCRT, the patient received maintenance immunotherapy with toripalimab 240 mg every 3 weeks. In June 2024, three months after completion of CCRT, follow-up imaging demonstrated new distant metastases, confirming disease progression. Progression was determined based on radiologic findings without histopathologic confirmation of the metastatic lesions. The patient subsequently received systemic therapy. The chronological sequence of clinical events and corresponding ctDNA-MRD dynamics is summarized in **Figure 4**.

## Discussion

Circulating tumor DNA is a tumor-derived fraction of cell-free DNA and has become an important tool across multiple solid tumors. It enables noninvasive assessment of minimal residual disease, supports treatment stratification, and allows real-time monitoring of therapeutic response (1, 4). Although MRD detection was first established in hematologic malignancies, recent advances in high-sensitivity assays have expanded its use in solid tumors (5, 6).

In several cancers, ctDNA-MRD has shown high accuracy for detecting recurrence, and growing evidence supports its role as a dynamic biomarker (7, 8). Research in HNSCC is more limited, but early findings are encouraging. ctDNA is detectable in most patients, and in HPV-related oropharyngeal cancer, changes in HPV ctDNA strongly correlate with remission and relapse (9, 10). Postoperative ctDNA positivity in HPV-negative HNSCC has also been linked to early recurrence (11, 12). However, ctDNA-MRD applications in the neoadjuvant setting—particularly during immunotherapy—remain scarce and understudied.

This case underscores the potential role of ctDNA-MRD as a sensitive biomarker for treatment monitoring in locally advanced HNSCC. Radiographic and endoscopic evaluations remain the cornerstone of response assessment in head and neck cancer, yet they may not reliably distinguish true tumor regression from post-treatment inflammation, fibrosis, or partial tumor replacement. In this patient, imaging suggested a deep response, with more than 70% tumor shrinkage and near-complete mucosal flattening, yet ctDNA-MRD levels rose rather than declined. This discordance highlights the blind spots of conventional imaging and illustrates how molecular progression may precede radiologic recurrence. Such discrepancies raise the possibility that some cases of “excellent imaging response” may actually represent undertreated systemic disease (13).

In the neoadjuvant setting, treatment evaluation directly influences subsequent management—whether to pursue surgery, definitive chemoradiation, or systemic therapy intensification. When imaging alone is used for assessment, patients with apparent major responses may proceed with local therapy despite unrecognized micrometastatic disease. The current case demonstrates that relying solely on radiologic response might have masked ongoing systemic progression. If ctDNA-MRD had been incorporated prospectively into decision-making, consideration could have been given to systemic treatment escalation or alternative strategies, rather than assuming near-complete disease control based on imaging alone. Importantly, ctDNA-MRD results in this case were generated within a non-interventional observational research framework and were blinded to the treating physicians; therefore, clinical decisions were made solely on standard radiologic and clinical assessments.

Accurate presurgical prediction of pathological complete response (pCR) can meaningfully inform treatment selection and may allow some patients to avoid unnecessary surgery, thereby improving quality of life. Yue (2) demonstrated the predictive value of ctDNA-MRD in esophageal cancer, yet comparable evidence in HNSCC is still lacking. Current data suggest that approximately 40% of patients with locally advanced head and neck squamous cell carcinoma may achieve pCR after neoadjuvant immunotherapy (14–18). Reliable identification of these responders would markedly optimize treatment pathways and could substantially improve outcomes for this patient group.

A methodological consideration relates to the choice between tumor-informed and tumor-agnostic (tumor-naïve) MRD assays (19, 20). Tumor-informed approaches, based on prior tumor tissue sequencing, generally offer higher specificity and lower risk of confounding from clonal hematopoiesis, but require sufficient tissue and longer turnaround time. Tumor-naïve assays allow broader applicability and faster implementation, though rigorous CHIP filtering is essential to ensure specificity. In HNSCC, where tissue availability and treatment timelines may limit feasibility, both strategies may have roles. Further studies are needed to determine the optimal approach in the neoadjuvant setting.

Several limitations should be acknowledged. Disease progression was determined radiologically without histopathologic confirmation, which precluded molecular comparison between metastatic tissue and baseline liquid biopsy findings. In addition, ctDNA-MRD was not prospectively integrated into therapeutic decision-making, and its potential impact on treatment selection remains hypothetical in this context. These considerations underscore the need for prospective studies evaluating molecular-guided strategies in the neoadjuvant setting.

## Conclusion

This case illustrated a clear mismatch between radiologic remission and ctDNA-MRD positivity in a patient with locally advanced hypopharyngeal squamous cell carcinoma undergoing neoadjuvant immunochemotherapy. The subsequent distant metastasis confirmed the ctDNA signal as an early indicator of occult progression. ctDNA-MRD monitoring may provide critical complementary information to guide therapeutic decisions beyond conventional imaging, and should be further validated in prospective studies involving neoadjuvant and consolidation settings in HNSCC.

## Data Availability

The data presented in the study are deposited in the National Genomics Data Center repository, accession number GSA-Human: HRA017376.
